# CMR Findings in COVID-19 Recovered Patients: A Review on Parametric Mapping, Feature-Tracking, and LGE

**DOI:** 10.31083/j.rcm2311355

**Published:** 2022-10-21

**Authors:** Mary Luz Mojica-Pisciotti, Roman Panovský, Tomáš Holeček, Lukáš Opatřil

**Affiliations:** ^1^International Clinical Research Center at St. Anne's University Hospital, 60200 Brno, Czech Republic; ^2^1st Department of Internal Medicine/Cardioangiology at St. Anne's University Hospital, 60200 Brno, Czech Republic; ^3^Faculty of Medicine, Masaryk University, 62500 Bohunice, Czech Republic; ^4^Department of Biomedical Engineering, Brno University of Technology, 61600 Brno, Czech Republic

**Keywords:** magnetic resonance image, SARS-CoV-2, parametric mapping, feature tracking, late gadolinium enhancement

## Abstract

On March 11, 2020, the World Health Organization raised the coronavirus disease 2019 (COVID-19) status to a pandemic level. The disease caused a global outbreak with devastating consequences, and a fair percentage of patients who have recovered from it continue experiencing persistent sequelae. Hence, identifying the medium and long-term effects of the COVID-19 disease is crucial for its future management. In particular, cardiac complications, from affected function to myocardial injuries, have been reported in these patients. Considering that cardiovascular magnetic resonance (CMR) imaging is the gold standard in diagnosing myocardial involvement and has more advantages than other medical imaging modalities, assessing the outcomes of patients who recovered from COVID-19 with CMR could prove beneficial. This review compiles common findings in CMR in patients from the general population who recovered from COVID-19. The CMR-based techniques comprised parametric mapping for analyzing myocardial composition, feature tracking for studying regional heart deformation, and late gadolinium enhancement for detecting compromised areas in the cardiac muscle. A total of 19 studies were included. The evidence suggests that it is more likely to find signs of myocardial injury in patients who recovered from COVID-19 than in healthy controls, including changes in T1 and T2 mapping relaxation times, affected strain, or the presence of late gadolinium enhancement (LGE) lesions. However, more than two years after the outbreak, there is still a lack of consensus about how these parameters may indicate cardiac involvement in patients who recovered from the disease, as limited and contradictory data is available.

## 1. Introduction

Coronavirus disease 2019 (COVID-19) is caused by the severe acute respiratory syndrome coronavirus 2 (SARS-CoV-2) that reached pandemic levels in March 2020. Up to July 22, 2022, the number of total cases compiled by the Center for Systems Science and Engineering at John Hopkins University reported 567,951,340 million cases and 6,380,835 deaths worldwide [1]. COVID-19 manifests mainly through respiratory symptoms. However, most organs are affected by the disease, including the cardiovascular system [2, 3, 4]. People infected with COVID-19 have a greater risk of experiencing cardiovascular disease, regardless of the disease severity and vaccination status [5]. They also are likely to suffer from myocarditis and myocardial injury [6, 7, 8, 9, 10].

Imaging modalities are fundamental in diagnosis, especially since COVID-19 requires a prompt response. For likely cardiovascular involvement, bedside echocardiography should be used as the first step for diagnosis and further referring [11]. Computed tomography might help identify pneumonia and rule out suspected causes of cardiac damage [11]. Nuclear medicine imaging could help diagnose pulmonary embolus, but its more comprehensive benefit is limited [11, 12]. On the other hand, cardiovascular magnetic resonance (CMR) has several advantages in assessing myocardial tissue and is the gold standard in diagnosing myocardial involvement [11, 13].

Besides being non-invasive, CMR encompasses advanced techniques that provide qualitative and quantitative information about cardiac function. Three of them are parametric mapping, feature-tracking (CMR-FT), and late gadolinium enhancement (LGE). CMR parametric mapping allows a quantitative analysis of regional myocardial composition based on changes in the relaxation times of water protons in the tissue (T1, T2, and T2*) and the extracellular volume (ECV) [14], aiding the quantification of myocardial disease processes. On the other hand, CMR-FT allows a quantitative analysis of regional heart deformation by myocardial strain assessment [15, 16]. Finally, LGE detects compromised areas in the cardiac muscle depending on the distribution of a contrast medium in the extracellular or intravascular space [17].

Two years after the COVID-19 pandemic began, the cardiovascular impact of this disease is better known. This work critically reviews the most relevant CMR imaging findings in parametric mapping, myocardial strain, and LGE in recovered COVID-19 patients from the general population.

## 2. Methods

For this literature review, we performed a comprehensive literature review in PubMed, Scopus, and Google Scholar, including the keywords “SARS-CoV-2” or “COVID-19” and “CMR” or “MRI” or “cardiac MR” and “MAPPING” or “LGE” or “T1” or “T2” or “STRAIN” or “FEATURE” or “TRACKING” or “CMR-FT”. We considered documents available until May 24, 2022. The search was limited to publications from and including 2020. We excluded case reports, reviews, editorials, comments, preprints, and documents in a different language than English. We selected scientific articles focused on adults. The search in PubMed and Scopus was done in R [18] (version 4.1.2; RStudio 2021.09.1 build 372, PBC, Boston, USA) using the “easyPubMed” and “rscopus” packages. The Google Scholar search was done manually, and the records were exported with their built-in tool. All the steps in the screening stage were performed in R. Duplications were removed based on the record’s digital object identifier (DOI).

The study eligibility was evaluated by verifying it contained reported CMR data on patients recovered from COVID-19, with a minimum number of subjects equal to 15 and at least 30 days from the diagnosis to the CMR study. Similarly, the type of study included retrospective, prospective, case-control, and research letters, provided they complied with the other criteria. We excluded reports considering specific populations, i.e., athletes.

We gathered the data into a comma-separated value file further processed in R, according to the fields: first author, year, study design, scanner type, cohort (sample size, participants, sex), age, days after COVID-19 diagnosis and CMR examination, left ventricle (LV) ejection fraction (EF), LV indexed end-diastolic (LVEDVI) volume, LV end-systolic volume (LVESVI), right ventricle (RV) EF, RV indexed end-diastolic volume (RVEDVI) and RV indexed end-systolic (RVESVI) volume, indexed LV stroke volume (LVSVI) and indexed RV stroke volume (RVSVI), LV mass index, T1 native, T1 enhanced, T2 mapping, ECV, global longitudinal strain (GLS), global circumferential strain (GCS), global radial strain (GRS) and LGE.

The effect size pooling between controls and recovered patients was determined with a random-effects model using standardized mean differences. This analysis included healthy volunteers and excluded other comparison groups. The confidence interval around the pooled effect was determined with Knapp-Hartung adjustments [19]. The heterogeneity was determined with Higgins & Thompson’s *$I^{2}$* statistic derived from Cochran’s Q [20] and the heterogeneity variance τ^2^ with the restricted maximum-likelihood estimator [21]. Outliers were identified and removed, and influence diagnostics were also performed. The analyses were performed using the R packages *meta*, *dmetar*, and *metafor*. For the studies reporting median (interquartile range) values, those were transformed to mean (standard deviation) using the Wan *et al*. [22] method.

## 3. CMR Findings in Recovered COVID-19 Patients

**Fig. 1.**
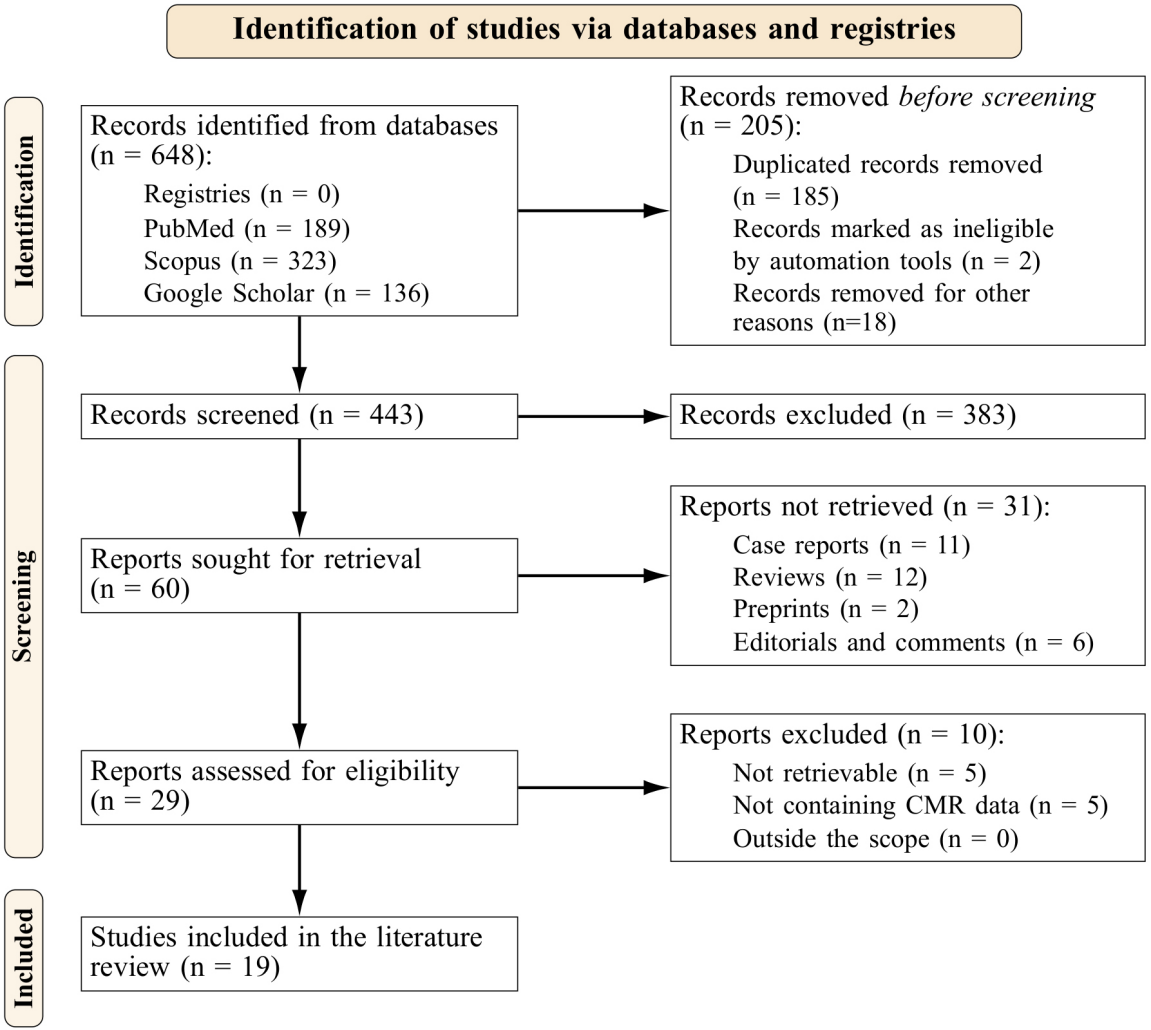
**Prisma flowchart**.

The PRISMA-S extension (Preferred Reporting Items for Systematic reviews and Meta-Analyses literature search extension) [23] flowchart is shown in Fig. 1. We identified 648 records, from which 60 were retrieved for further analysis. Following the eligibility assessment, nineteen studies were finally included in this review [4, 24, 25, 26, 27, 28, 29, 30, 31, 32, 33, 34, 35, 36, 37, 38, 39, 40, 41]; their characteristics are shown in Table 1 (Ref. [4, 24, 25, 26, 27, 28, 29, 30, 31, 32, 33, 34, 35, 36, 37, 38, 39, 40, 41]).

**Table 1.** **General description of the included studies**.

Author, year	Study design	N (m/f)	Cohort description	Age ${\mathrm{(y)}}^{1}$
Field strength = 1.5 T				
Altay [24], 2021	Retrospective, SC	15 (8/7)	Patients who recovered from COVID-19	15 ± 8
		20 (12/8)	Controls	20 ± 12
Breitbart *et al*. [25], 2021	Prospective, SC	56 (26/30)	Post-COVID-19 patients with no history of previous heart disease	45.7 ± 12.2
Haberka *et al*. [26], 2022 (L)	Prospective, MC (5 centers)	300	Patients who recovered from COVID-19 with myocarditis	45.6 ± 12
		150	Patients with non-COVID-19 myocarditis	42.8 ± 14
Kotecha *et al*. [28], 2021	Retrospective, MC (6 hospitals)	148 (104/44)	Patients who recovered from COVID-19	64 ± 12
		40 (28/12)	Patients without clinical suspicion of myocardial injury (historical control group)	64 ± 9
		40 (23/17)	Healthy volunteers	49 ± 6
Kravchenko *et al*. [29], 2021	Prospective, SC	41 (18/23)	Patients with chronic COVID-19 syndrome	39 ± 13
		42 (26/16)	Controls	40 ± 16
Myhre *et al*. [32], 2021	Prospective, SC	58 (34/24)	COVID-19 survivors	56 (49 to 70)
		32 (14/18)	Healthy controls	69 (69 to 69)
Ng *et al*. [33], 2020 (L)	Retrospective, SC	16 (9/7)	Patients who recovered from COVID-19	68 (53 to 69)
		15	Healthy volunteers	
Tanacli *et al*. [36], 2021	Prospective, SC	32 (19/13)	Patients with persistent cardiac symptoms after a COVID-19 infection	48 ± 14
		22 (17/5)	Patients with acute non-COVID-19-related myocarditis	32 ± 15
		16 (8/8)	Healthy volunteers	24 ± 5
Thornton *et al*. [37], 2021	Prospective, MC (3 centers)	90 (75/15)	Recovered post-COVID-19 patients	64 (54 to 71)
		90 (73/17)	Controls	60 (49 to 68)
		27 (14/13)	Healthy volunteers	33 (30 to 42)
Urmeneta Ulloa *et al*. [38], 2021	Prospective, SC	57 (46/11)	Post-COVID-19 patients	59 ± 15
		20	Healthy controls	
Wojtowicz *et al*. [40], 2021 (L)	Prospective, SC	50 (20/30)	Consecutive patients who recovered from COVID-19 with persistent cardiac symptoms	47.3 ± 10.1
Zhang *et al*. [41], 2022	Prospective, SC	44 (16/28)	Patients who recovered from delta variant COVID-19	51 (39 to 62)
		25 (14/11)	Healthy controls	44 (39 to 51)
Field strength = 1.5 T/3.0 T				
Li D. *et al*. [30], 2021	Prospective, SC	21 (15/6)	Patients who recovered from COVID-19 with multisystem inflammatory syndrome (MIS)-myocarditis	14 (8 to 20)
		19 (11/8)	Patients who recovered from COVID-19 with non-MIS myocarditis	24 (20 to 50)
Field strength = 3.0 T				
Li X. *et al*. [31], 2021	Prospective, SC	24 (12/12)	Patients who recovered from moderate COVID-19	52 ± 10
		16 (12/4)	Patients who recovered from severe COVID-19	57 ± 15
		24 (16/8)	Healthy controls	50 ± 15
Huang *et al*. [27], 2020	Retrospective, SC	15 (4/11)	Patients who recovered from COVID-19 with conventional CMR findings	39 (29 to 49)
		11 (6/5)	Patients who recovered from COVID-19 without conventional CMR findings	37 (34 to 39)
		20 (7/13)	Healthy controls	40 (29 to 50)
Pan *et al*. [34], 2021	Prospective, SC	21 (10/11)	Patients who recovered from COVID-19	36 (31 to 47)
		20 (8/12)	Healthy controls	50 (32 to 61)
Puntmann *et al*. [35], 2020	Prospective, SC	100 (53/47)	Unselected patients who recovered from COVID-19	49 ± 14
		50 (25/25)	Age- and sex-matched healthy volunteers	48 ± 16
		57 (28/29)	Risk factor-matched patients	49 ± 13
Raman *et al*. [4], 2021	Prospective, SC	58 (34/24)	Patients who recovered from COVID-19	55.4 ± 13.2
		30 (18/12)	Controls	53.9 ± 12.3
Wang *et al*. [39], 2021	Prospective, SC	13 (4/9)	Patients who recovered from COVID-19 with LGE	53.2 ± 14.5
		31 (15/16)	Patients who recovered from COVID-19 without LGE	45.2 ± 12.3
		31 (19/12)	Healthy controls	47.1 ± 11.0

f, female; m, male; (L), letter; LGE, late gadolinium enhancement; MC, multicenter; MIS, multisystem inflammatory syndrome; N, number of participants; SC, single-center; T, Tesla; y, year. ^1^Results are presented as mean (standard deviation) or median (interquartile range).

The total number of included subjects was 2007, from which 1217 were patients who recovered from COVID-19, 502 were controls or healthy volunteers, and 288 were from other comparison groups, i.e., patients with myocarditis or suspicion of myocardial injury. The mean age of participants was 45.9 ± 13.1 years (recovered patients 47.2 ± 13.5 years, controls 44.8 ± 12.9 years, other comparison groups 43.5 ± 14.0 years). Table 1 summarizes cohort details in each study.

### 3.1 CMR Parameters

COVID-19 has been linked to myocardial inflammation and myocardial injury [7, 8, 42] following the established CMR criteria for such a diagnosis. The updated Lake Louis criteria include parametric mapping for diagnosing myocardial inflammation: while native T1 mapping and ECV are linked to myocardial injury, T2 mapping is linked to myocardial edema [43]. Extensive works about the connection between COVID-19 and myocarditis can be found elsewhere [6, 7, 9, 42, 44, 45] and not be described here.

Other CMR-derived parameter findings in COVID-19 recovered subjects compared to healthy volunteers include lower left ventricular (LV) EF and right ventricular (RV) EF [27, 30, 34, 35, 36]. In most studies, the LV end-diastolic volume (EDV) index was also lower [26, 28, 37, 41] except for one, where it was higher in the recovered subjects [35]. Similarly, they exhibited a lower RV stroke volume (SV) index [34]. Additionally, abnormal findings have been reported in 58% (N = 26, myocardial edema and LGE) [27], 71% (N = 21, decreased left ventricular ejection fraction (LVEF) and right ventricular ejection fraction (RVEF), raised parametric mapping values) [34] and 78% of the included subjects (N = 100, raised parametric mapping values, LGE or pericardial enhancement) [35].

In other studies, patients who recovered from COVID-19 had lower LVEF and LVEDV than patients with non-COVID-19 myocarditis [26] and LVEF and myocardial mass than risk-factor matched patients; a higher LVEDVI was reported for this subgroup [35]. Similarly, lower LVEF and LVSVI were found in patients who recovered from COVID-19 and had multisystem inflammatory syndrome (MIS)-myocarditis than those with non-MIS myocarditis. RVEF was also significantly lower in recovered subjects compared to historical control [28] and risk-factor matched groups [35]. Finally, patients with acute non-COVID-19 related myocarditis were found to have a higher RVESVI than patients with persistent cardiac symptoms after a COVID-19 infection [36].

### 3.2 Parametric Mapping

The available native T1 relaxation times and ECV values are shown in Table 2 (Ref. [4, 25, 27, 28, 29, 30, 31, 32, 33, 34, 35, 36, 37, 38, 39, 41]). The median time from diagnosis to the CMR examination of patients recovered from COVID-19 was 74.25 days (interquartile range (IQR) 50.75 to 98.75). The reference values of T1 native, T2, and ECV depend on many factors, including the sequence type and the scanner; each center typically adjusts and provides its reference ranges. Nonetheless, as a general guide, normal values can be found in the following ranges: T1 native: 885 ms to 1073 ms (1.5 T) and 964 ms to 1290 ms (3.0 T); T2: 42 ms to 65 ms (1.5 T) and 37 ms to 58 ms (3.0 T); and ECV: 17% to 33% (1.5 T) and 16% to 36% (3.0 T) [46].

**Table 2.** **Reported T1 native and ECV values**.

Author, year	Cohort description	Native T1 ${\mathrm{(ms)}}^{1}$	ECV ${\mathrm{(\%)}}^{1}$
Field strength = 1.5 T			
Breitbart *et al*. [25], 2021	56 post-COVID-19 patients with no history of previous heart disease	1016.0 ± 28.2	27.5 ± 3.4
Kotecha *et al*. [28], 2021	148 patients recovered from COVID-19	1033 ± 41	
	40 patients without clinical suspicion of myocardial injury (historical control group)	1028 ± 35	
	40 healthy volunteers	1008 ± 35	
Kravchenko *et al*. [29], 2021	41 patients with chronic COVID-19 syndrome	978 ± 23	24.1 ± 2.3
	42 controls	971 ± 25	25.1 ± 2.6
Myhre *et al*. [32], 2021	58 COVID-19 survivors	1006 ± 31	24.8 ± 2.8
	32 healthy controls	993 ± 29	25.9 ± 2.1
Ng *et al*. [33], 2020 (L)	16 patients who recovered from COVID-19	1209 (1164 to 1219)	
	15 healthy volunteers	1158 (1190 to 1208)	
Tanacli *et al*. [36], 2021	32 patients with persistent cardiac symptoms after a COVID-19 infection	1271 ± 50	25 ± 4
	22 patients with acute non-COVID-19-related myocarditis	1352 ± 113	30 ± 8
	16 healthy volunteers	1236 ± 21	26 ± 4
Thornton *et al*. [37], 2021	90 recovered post-COVID-19 patients	1032 (1008 to 1061)	26 (23 to 29)
	90 controls		
	27 healthy volunteers	1008 ± 35	
Urmeneta Ulloa *et al*. [38], 2021	57 post-COVID-19 patients	996.4 ± 43.9	26.6 ± 3.1
	20 healthy controls	981.5 ± 21.2	
Zhang *et al*. [41], 2022	44 patients who recovered from delta variant COVID-19	1318.8 ± 55.5	26.2 ± 4.0
	25 healthy controls	1282.9 ± 38.1	26.7 ± 1.9
Field strength = 1.5 T/3.0 T			
Li D *et al*. [30], 2021	21 patients who recovered from COVID-19 with multisystem inflammatory syndrome (MIS)-myocarditis	${\mathrm{Z-score}}^{2}$: 3.3 (2.2 to 5.9)	33 (28 to 35)
	19 patients who recovered from COVID-19 with non-MIS myocarditis	${\mathrm{Z-score}}^{2}$: 1.5 (0.4 to 3.6)	31 (27 to 33)
Field strength = 3.0 T			
Huang *et al*. [27], 2020	15 patients who recovered from COVID-19 with conventional CMR findings	1271 (1243 to 1298)	28.2 (24.8 to 36.2)
	11 patients who recovered from COVID-19 without conventional CMR findings	1237 (1216 to 1262)	24.8 (23.1 to 25.4)
	20 healthy controls	1224 (1217 to 1245)	23.7 (22.2 to 25.2)
Li X *et al*. [31], 2021	24 patients who recovered from moderate COVID-19	1134.5 (1114.0 to 1210.0)	29.7 (28.0 to 32.9)
	16 patients who recovered from severe COVID-19	1140 (1062.8 to 1183.8)	31.4 (29.3 to 34.0)
	25 healthy controls	1138.1 (1092.9 to 1166.2)	25.0 (23.7 to 26.0)
Pan *et al*. [34], 2021	21 patients who recovered from COVID-19	1208.4 ± 64.2	
	20 healthy controls	1213.6 ± 61.7	
Puntmann *et al*. [35], 2020	100 unselected patients who recovered from COVID-19	1125 (1099 to 1157)	
	50 age- and sex-matched healthy volunteers	1082 (1067 to 1097)	
	57 risk factor-matched patients	1111 (1098 to 1124)	
Raman *et al*. [4], 2021	58 patients who recovered from COVID-19	1173.1 ± 33.6	30.1 (27.2 to 31.4)
	30 controls	1150.2 ± 32.4	29.4 (27.1 to 30.7)
Wang *et al*. [39], 2021	13 patients who recovered from COVID-19 with LGE	1286 ± 60	
	31 patients who recovered from COVID-19 without LGE	1253 ± 55	
	31 healthy controls	1122 ± 57	

(L), letter; T, Tesla. ^1^Results are presented as mean (standard deviation) or median (interquartile range). ^2^Z-score is calculated from the formula: (subject mean-control mean)/(control standard deviation).

Studies including patients who recovered from COVID-19 reported increased [4, 27, 28, 33, 35, 36], slightly increased [29, 32, 38, 39, 41] or similar [31, 34] T1 native mapping values compared to controls. In particular, Kotecha *et al*. [28], Puntmann *et al*. [35], and Thornton *et al*. [37] found significantly higher T1 native mapping values in large groups of recovered subjects (148, 100 and 90, respectively) compared to different control groups, including patients without myocardial injury, healthy volunteers or controls, and risk factor-matched controls [26, 28, 35, 37]. In addition, patients who recovered from COVID-19 and had MIS-myocarditis had higher T1 native values than those with non-MIS myocarditis [30]. Other studies assessing slightly higher or similar T1 native values did not reach statistical significance [29, 31, 32, 34, 38, 39].

The pooled effect size for T1 native values from studies reporting values measured at 1.5 T was 0.59 (95% CI 0.25 to 0.94) and was statistically significant (*p* = 0.0054), with moderate heterogeneity. On the other hand, the equivalent pooled effect size resulting from studies measuring at 3.0 T was 1.96 (95% CI 0.06 to 3.86) and significant (*p* = 0.0452), with high between-study heterogeneity. This result means a significant difference between the T1 native values of recovered patients and controls in all studies, favoring increased values for the patients. The corresponding forest plots are shown in** Supplementary Fig. 1**.

Fewer studies reported significantly higher ECV in patients who recovered from COVID-19, particularly those with a severe disease manifestation [27, 31]. Other authors found similar values between recovered subjects and controls [4, 29, 30, 32, 41]. The pooled effect size for ECV measured at 1.5 T was –0.32 (95% CI –0.54 to –0.11) and was statistically significant (*p* = 0.0169). For values measured at 3.0 T, the pooled effect size was 2.70 (95% CI –2.98 to 8.38) and non-significant (*p* = 0.1771). The corresponding forest plots are shown in **Supplementary Fig. 2**.

The available T2 relaxation times are shown in Table 3 (Ref. [4, 27, 28, 29, 32, 33, 35, 38, 41, 47]) Here, increased or raised values have been consistently reported in patients who recovered from COVID-19 [27, 34, 35, 36, 38, 47]. One study reported a significantly lower T2 in this group than healthy volunteers and a historical control group [28]. The pooled effect size for T2 from studies reporting values measured at 1.5 T was non-significant (0.34 (95% CI –0.35 to 1.03), *p* = 0.2756) and had substantial heterogeneity. For studies reporting values measured at 3.0 T, the pooled effect size was significant (0.87 (95% CI 0.09 to 1.63), *p* = 0.0372) and had high heterogeneity. The corresponding forest plots are shown in **Supplementary Fig. 3**.

**Table 3.** **Reported T2 mapping values and other signs of edema and pericarditis**.

Author	Cohort description	T2 ${\mathrm{(ms)}}^{1}$	Visible edema ${\mathrm{(N (\%))}}^{1}$	Pericardial effusion ${\mathrm{(N (\%))}}^{1}$	Pericardial enhancement ${\mathrm{(N (\%))}}^{1}$
Field strength = 1.5 T					
Kotecha* et al*. [28], 2021	148 patients who recovered from COVID-19	46 ± 3	0 (0%)	8 (5%)	
	40 patients without clinical suspicion of myocardial injury (historical control group)	47 ± 3			
	40 healthy volunteers	48 ± 2			
Kravchenko* et al*. [29], 2021	41 patients with chronic COVID-19 syndrome	53 ± 2	0 (0%)	1 (2%)	
	42 controls	52 ± 2	0 (0%)		
Myhre* et al*. [32], 2021	58 COVID-19 survivors	51.6 ± 2.8	0 (0%)		0 (0%)
	32 healthy controls	52.7 ± 4.0			
Ng* et al*. [33], 2020 (L)	16 patients who recovered from COVID-19	52 (50 to 56)		0 (0%)	
	15 healthy volunteers	48.2 (41.5 to 54.8)			
Urmeneta Ulloa* et al*. [38], 2021	57 post-COVID-19 patients	50.9 ± 4.3	2 (3.5%)		
	20 healthy controls	48.0 ± 1.9			
Zhang* et al*. [41], 2022	44 patients who recovered from delta variant COVID-19	47.6 ± 4.2	5 (11%)	4 (9%) [≥5 mm]	2 (5%)
	25 healthy controls	47.4 ± 2.2	0 (0%)		0 (0%)
Field strength = 3.0 T					
Huang* et al*. [27], 2020	15 patients who recovered from COVID-19 with conventional CMR findings	42.7 ± 3.1	26 (54%)	7 (50%)	
	11 patients who recovered from COVID-19 without conventional CMR findings	38.1 ± 2.4			
	20 healthy controls	39.1 ± 3.1			
Pan* et al*. [47], 2021	21 patients who recovered from COVID-19	49.2 (46.1 to 54.6)	0 (0%)		
	20 healthy controls	48.3 (45.2 to 51.7)			
Puntmann* et al*. [35], 2020	100 unselected patients who recovered from COVID-19	38.2 ± 2.0		20 (20%) [>10 mm]	22 (22%)
	50 age- and sex-matched healthy volunteers	35.7 ± 1.5		0 (0%)	
	57 risk factor-matched patients	36.4 ± 1.6		4 (7%) [>10 mm]	
Raman* et al*. [4], 2021	58 patients who recovered from COVID-19	41.8 ± 2.2		1 (1.9%) [>10 mm]	
	30 controls	41.1 ± 2.3		0 (0%)	

(L), letter; N, number of subjects; T, Tesla. ^1^Results are presented as mean (standard deviation) or median (interquartile range).

Generally, T2 values result higher with increased water content in the myocardium, a feature of both ischemic and non-ischemic cardiomyopathies commonly associated with acute myocardial inflammation [14, 48]. With image quality and reproducibility as the limiting factors of its wider clinical adoption, T2 mapping emerged with its quantitative nature and higher robustness [14]. Though in the case of myocarditis, T1 mapping has better diagnostic accuracy and positive and negative predictive values [49, 50], T2 mapping seems superior for assessing this disease activity in patients [51]. Therefore, in patients with increased T2 values, an active inflammatory process is expected. In the case of lower T2 values [28], these have been reported in healthy males compared to females [52], with an unknown underlying reason for this phenomenon.

CMR parametric mapping in patients who recovered from COVID-19 revealed higher T1 native values than in control groups. In most studies, recovered patients also exhibited increased or raised T2 values [4, 27, 29, 34, 35, 38, 41]. Finally, ECV was reported in a few studies [4, 25, 27, 29, 30, 31, 32, 36, 37, 38, 41], and their pooled effect suggests that patients who recovered from COVID-19 have similar values to controls.

### 3.3 Cardiovascular Magnetic Resonance – Feature Tracking (CMR-FT)

CMR-FT is an emerging tool for quantitative analysis of regional heart deformation [15, 16]. It is based on optical flow, a technique used to track the movement of individual pixels in a series of images [16]. Such a process allows contouring different heart regions to follow their movement and measure parameters related to their deformation, which can offer insight into underlying cardiac problems. One of those parameters is the strain, which, in the LV, describes regional changes related to the shortening, thickening, or lengthening of the myocardium. The assessment is usually done through the GCS, GRS, and GLS. Several studies have found that strain may serve as a more efficient marker of contractile dysfunction than other clinical ones [53, 54, 55].

CMR-FT assessment in patients who recovered from COVID-19 showed that their left ventricle global longitudinal strain (LVGLS) is lower than controls [31, 32, 36, 41]. One study reported similar values for patients who recovered from COVID-19 without LGE and controls and significantly lower values in the case of patients with LGE [39]. Regarding the left ventricle global radial strain (LVGRS), no differences were found in any of the selected studies that reported this parameter [31, 38, 39, 41]. Left ventricle global circumferential strain (LVGCS), on the other hand, compared to healthy controls, was significantly lower in recovered patients with LGE [39], the delta variant [41], or acute non-COVID-19-related myocarditis [36]. One study reported that the right ventricle global circumferential strain (RVGCS) and the right ventricle global longitudinal strain (RVGLS) were significantly lower in recovered patients with LGE than those without LGE and healthy controls, and the right ventricle global radial strain (RVGRS) had no changes [39]. Finally, RVGLS was also lower in patients who recovered from COVID-19 compared to controls but higher than in patients with acute non-COVID-19-related myocarditis [36]. The data summary of LV and RV strains is shown in **Supplementary Table 1**.

### 3.4 Late Gadolinium Enhancement (LGE)

LGE is the gold standard and most validated technique for assessing myocardial scar, inflammation, or necrosis [17, 56]. This technique allows differentiating normal and abnormal myocardium based on their T1 longitudinal relaxation times using an extracellular gadolinium-based contrast agent. In normal tissue, the cell membrane is compact, and the contrast agent is washed out quickly, resulting in a low concentration of gadolinium and a longer T1. In abnormal tissue, the contrast agent accumulates, shortening T1 [57, 58]. Therefore, affected areas appear hyperintense in an LGE-CMR image depending on the tissue’s physiological properties.

LGE data from the selected reports are summarized in Table 4 (Ref [4, 24, 25, 26, 27, 28, 30, 31, 33, 35, 36, 37, 38, 39, 40, 41]). Kotecha *et al*. [28] found significant positive LGE in 49% of the patients who recovered from COVID-19, Thornton *et al*. [37] reported it in 56%, and Puntmann *et al*. [35] found 32% (myocardial) and 22% (pericardial). An author reported LGE with non-specific distribution and appearance [24], while others found subepicardial, subendocardial, and intramyocardial [25, 28, 41], myocarditis-like [4, 26], and non-ischemic [33, 38] patterns. Generally, LGE patterns can help differentiate between ischemic and non-ischemic myocardial injury. Ischemic injury tends to cause LGE, usually subendocardial or transmural [59, 60]. Non-ischemic, on the other hand, appears typically at the epicardium, mid-wall, or insertion points [60, 61]. In addition, the ischemic injury pattern should be consistent with the vascular supply from the main coronary arteries, which in some cases, may be anomalous, and their anatomy might vary [60, 62].

**Table 4.** **Reported LGE data**.

Author, year	Notes
Altay [24], 2021	7 (47%) patients who recovered from COVID-19 had LGE with non-specific distribution and appearance. Further data regarding CMR findings show significant differences in LVEF, RVEF, and LVSV between recovered patients with LGE, without LGE, and controls.
Breitbart *et al*. [25], 2021	7 (12.5%) post-COVID-19 patients with no history of previous heart disease had LGE. The patterns were subepicardial: 5 (8.9%), subendocardial: 1 (1.8%), and intramyocardial: 1 (1.8%).
Haberka *et al*. [26], 2022 (L)	4.2 ± 4.4 (injured segments) were detected in patients who recovered from COVID-19 with myocarditis. Also, 15% had pericarditis, and 51% had LGE with a myocarditis-like pattern. 4.3 ± 2.9 (injured segments) were detected in patients with non-COVID-19 myocarditis; 7% had pericarditis.
Huang *et al*. [27], 2020	Myocardial edema was found in 14 of 26 patients who recovered from COVID-19 (54%); 7 of 14 had positive LGE, and 7 of 14 had small pericardial effusion. One patient had LGE but no obvious myocardial edema.
Kotecha *et al*. [28], 2021	70 (49%) patients recovered from COVID-19 had LGE with subendocardial or transmural: 28 (16%), mid-myocardial: 16 (11%), subepicardial: 31 (22%) patterns. 18 (45%) patients without clinical suspicion of myocardial injury (historical control group) had LGE with subendocardial or transmural: 10 (15%), mid-myocardial: 6 (15%), and subepicardial: 2 (5%) patterns.
Li D. *et al*. [30], 2021	The LGE burden was 5.9 (3.1 to 11.6) % in patients who recovered from COVID-19 with multisystem inflammatory syndrome (MIS)-myocarditis, with LGE frequently located at the septum; 63.3% with LGE were either anteroseptal or inferoseptal segments. For patients who recovered from COVID-19 with non-MIS myocarditis, the LGE burden was 6.6 (3.8 to 8.0) %, with LGE in the inferior half of the myocardium; 27.8% were septal segments.
Li X. *et al*. [31], 2021	1 (6%) patient who recovered from severe COVID-19 had LGE in the mid-inferior wall.
Ng *et al*. [33], 2020 (L)	3 (19%) patients who recovered from severe COVID-19 had non-ischemic LGE.
Puntmann *et al*. [35], 2020	In unselected patients recovered from COVID-19, LGE pattern was myocardial: 32 (32%), non-ischemic: 20 (20%), and pericardial 22 (22%). In risk factor-matched patients, it was myocardial: 9 (17%), non-ischemic: 4 (7%), and pericardial 8 (14%).
Raman *et al*. [4], 2021	11.50% of patients who recovered from COVID-19 had LGE with a myocarditis pattern, and 1.9% had a myocardial infarction. 7.4% of controls had LGE with a myocarditis pattern.
Tanacli *et al*. [36], 2021	LGE in patients with persistent cardiac symptoms after a COVID-19 infection was ischaemic: 1 (3%), non-ischemic: 5 (16%), and pericardial: 3 (10%). In patients with acute non-COVID-19-related myocarditis, it was ischaemic: 3 (14%), non-ischaemic: 19 (86%), and pericardial: 4 (18%).
Thornton *et al*. [37], 2021	50 (56%) of patients with persistent cardiac symptoms after a COVID-19 infection had LGE with an infarct pattern: 15 (17%), non-ischemic: 31 (34%), and mixed pattern: 4 (4.4%).
Urmeneta Ulloa *et al*. [38], 2021	15 (26.3%) patients who recovered from COVID-19 had LGE with non-ischaemic: 11 (19.3%), ischaemic: 2 (3.5%), and pericardial: 2 (3.5%) patterns.
Wang *et al*. [39], 2021	13 (29.5%) patients who recovered from COVID-19 had all LGE lesions in the middle myocardium and/or sub-epicardium. The most frequently involved walls were the inferior and inferior-lateral of the basal segment.
Wojtowicz *et al*. [40], 2021 (L)	30 (60%) patients who recovered from COVID-19 had most LGE lesions located at the inferolateral (76%, n = 23) and inferior (43%, n = 13) segments.
Zhang *et al*. [41], 2022	4 (9%) patients recovered from delta variant COVID-19 had LGE in the mid-wall: 1 (2%), subepicardial: 3 (7%).

(L), letter.

LGE in the myocardium has been well documented as a negative predictive factor in many cardiac conditions, such as dilated and hypertrophic cardiomyopathy [63]. It has also been linked to higher mortality in cardiac amyloidosis [64]. Gutman *et al*. [65] showed that including LGE assessment by CMR can lead to a better selection of patients with an indication for implantable cardioverter-defibrillator implantation.

On the other hand, although the general prognosis of patients with myocarditis seems rather good [66], it tends to have a very variable course ranging from complete remission to severe complications [67]. Some studies have also shown a worse prognosis in patients with positive LGE in myocarditis or myocardial inflammation [68]. However, the data are limited, and persistent LGE was reported in over 50% of these patients in a one-year CMR follow-up [66].

While no long-term follow-up of COVID-19 patients is available, it is feasible to assume a worse prognosis for patients with LGE than those without LGE or healthy controls, considering already published data. Such worsening could involve a higher incidence of heart failure or an increased chance of sudden cardiac death, possibly reducing the life expectancy in these patients.

## 4. Limitations

Reports included in this review had limitations related to sample size [24, 25, 27, 31, 34, 39, 41], study design [25, 30, 37, 39, 41], CMR-data related effects [30, 31, 38], lack of follow-up data [27, 30, 39, 41], clinical validation of the findings [29] and validation in other cohorts [35], and a previous CMR baseline exam [24, 32, 39]. However, selection bias [24, 25, 27, 28, 29, 30, 32, 34, 37, 38, 41] and other biases [24, 28, 29, 30, 32, 35, 36, 37], including unblinded analyses, guidelines recommendations, the possibility of cardiac MR findings before the SARS-CoV-2 infection, survivor bias, and age differences, were the most common limitations. Finally, almost 90% of the included articles were done during the critical pandemic phases, reflecting constraints due to the worldwide increased demand for medical and clinical resources.

## 5. Conclusions 

More than two years after the outbreak, there is still a lack of consensus about how CMR-derived indicators may signal cardiac involvement in patients who recovered from COVID-19. However, most of the selected articles in this review report some extent of myocardial injury in these patients, regardless of conflicting or ambiguous data. With hundreds of millions of cases to date, and a growing number of cases, myocardial involvement could present a threat and heavy burden for healthcare systems worldwide.

Extensive, comprehensive multicenter prospective studies are still needed to understand how myocardial involvement affects patients who recovered from COVID-19. With new variants seemingly more contagious, though with a decreased rate of hospitalizations and mortality, further studies must be performed, ranging from asymptomatic to severe cases. In addition, considering that signs of myocardial injury are already linked with poor prognosis in different cardiac diagnoses, follow-up studies of these patients, especially those with LGE, could aid the early identification of persistent or developing cardiac pathologies.

## Supplementary Material

Supplementary material associated with this article can be found, in the online version, at https://doi.org/10.31083/j.rcm2311355.
