# Supplementary material for: CMR Findings in COVID-19 Recovered Patients: A Review on Parametric Mapping, Feature-Tracking, and LGE
Source: Rev Cardiovasc Med. 2022 Oct 21;23(11):355. doi: 10.31083/j.rcm2311355 (PMC11269062; doi:10.31083/j.rcm2311355)
Supplement: Supplementary file 1 [file 2153-8174-23-11-355-s1.docx]

****SUPPLEMENTARY DATA****

**CMR FINDINGS IN COVID-19 RECOVERED PATIENTS: A REVIEW ON PARAMETRIC MAPPING, FEATURE-TRACKING, AND LGE**

Mary Luz Mojica-Pisciotti, PhD.^1^, Roman Panovský, MUDr. PhD.^1,2,3^, Tomáš Holeček, Ing.^1,4^, Lukáš Opatřil, MUDr.^1,2,3^

Supplementary Table 1. Reported LV and RV strain data.

| **Author, year** | **N** | **Cohort description (sex)** | **LVGLS (%)** | **LVGRS (%)** | **LVGCS (%)** | **RVGLS (%)** | **RVGRS (%)** | **RVGCS (%)** |
| --- | --- | --- | --- | --- | --- | --- | --- | --- |
| **Field strength = 1.5 T** | | |  |  |  |  |  |  |
| Myhre *et al*. [32], 2021 | 58 | COVID-19 survivors | –16.3 ± 2.1 |  | –18.8 ± 3.2 |  |  |  |
|  | 32 | Healthy controls | –16.4 ± 2.1 |  | –18.9 ± 3.0 |  |  |  |
| Tanacli *et al*. [36], 2021 | 32 | Patients with persistent cardiac symptoms after a COVID-19 infection | –24.2 ± 4.4 |  | –32.0 ± 7.2 | –28.2 ± 7.8 |  |  |
|  | 22 | Patients with acute non-COVID-19-related myocarditis | –20.1 ± 7.0 |  | –24.0 ± 9.3 | –27.1 ± 7.3 |  |  |
|  | 16 | Healthy volunteers | –26.0 ± 2.1 |  | –31.5 ± 4.4 | –29.7 ± 7.1 |  |  |
| Urmeneta Ulloa *et al*. [38], 2021 | 57 | Post-COVID-19 patients |  | 32.3 ± 8.1 | 18.6 ± 3.3 |  |  |  |
|  | 20 | Healthy controls |  | 33.6 ± 7.1 | 19.2 ± 2.1 |  |  |  |
| Zhang *et al*. [41], 2022 | 44 | Patients recovered from delta variant COVID-19 | –22.2 ± 2.8 | 100.6 ± 34.3 | –20.7 ± 6.8 |  |  |  |
|  | 25 | Healthy controls | –24.6 ± 2.0 | 107.0 ± 30.2 | –24.3 ± 2.6 |  |  |  |
| **Field strength = 3.0 T** | | |  |  |  |  |  |  |
| Li X. *et al*. [31], 2021 | 24 | Patients recovered from moderate COVID-19 | –12.5 (–10.7 to –15.5) | 29.8 (9.8 to 40.6) | –20.8 (–16.9 to –22.7) |  |  |  |
|  | 16 | Patients recovered from severe COVID-19 | –12.5 (–8.7 to –15.4) | 20.5 (11.4 to 41.3) | –22.7 (–14.5 to –24.7) |  |  |  |
|  | 25 | Healthy controls | –15.4 (–14.6 to –17.6) | 30.4 (25.0 to 35.8) | –22.4 (–20.4 to –23.4) |  |  |  |
| Wang *et al*. [39], 2021 | 13 | Patients recovered from COVID-19 with LGE | –11.9 ± 3.9 | 40.5 ± 18.7 | –15.1 ± 10.3 | –7.8 ± 4.0 | 31.4 ± 14.5 | –9.4 ± 3.4 |
|  | 31 | Patients recovered from COVID-19 without LGE | –12.7 ± 3.6 | 46.3 ± 23.0 | –16.7 ± 6.9 | –12.9 ± 3.0 | 28.8 ± 11.1 | –12.1 ± 4.0 |
|  | 31 | Healthy controls | –13.1 ± 2.8 | 39.8 ± 15.8 | –19.4 ± 3.0 | –11.3 ± 3.9 | 34.4 ± 10.4 | –12.9 ± 4.3 |

GCS, global circumferential strain; GLS, global longitudinal strain; GRS, global radial strain; LV, left ventricle; RV, right ventricle.

All numerical results are presented as mean (standard deviation) or median (interquartile range).


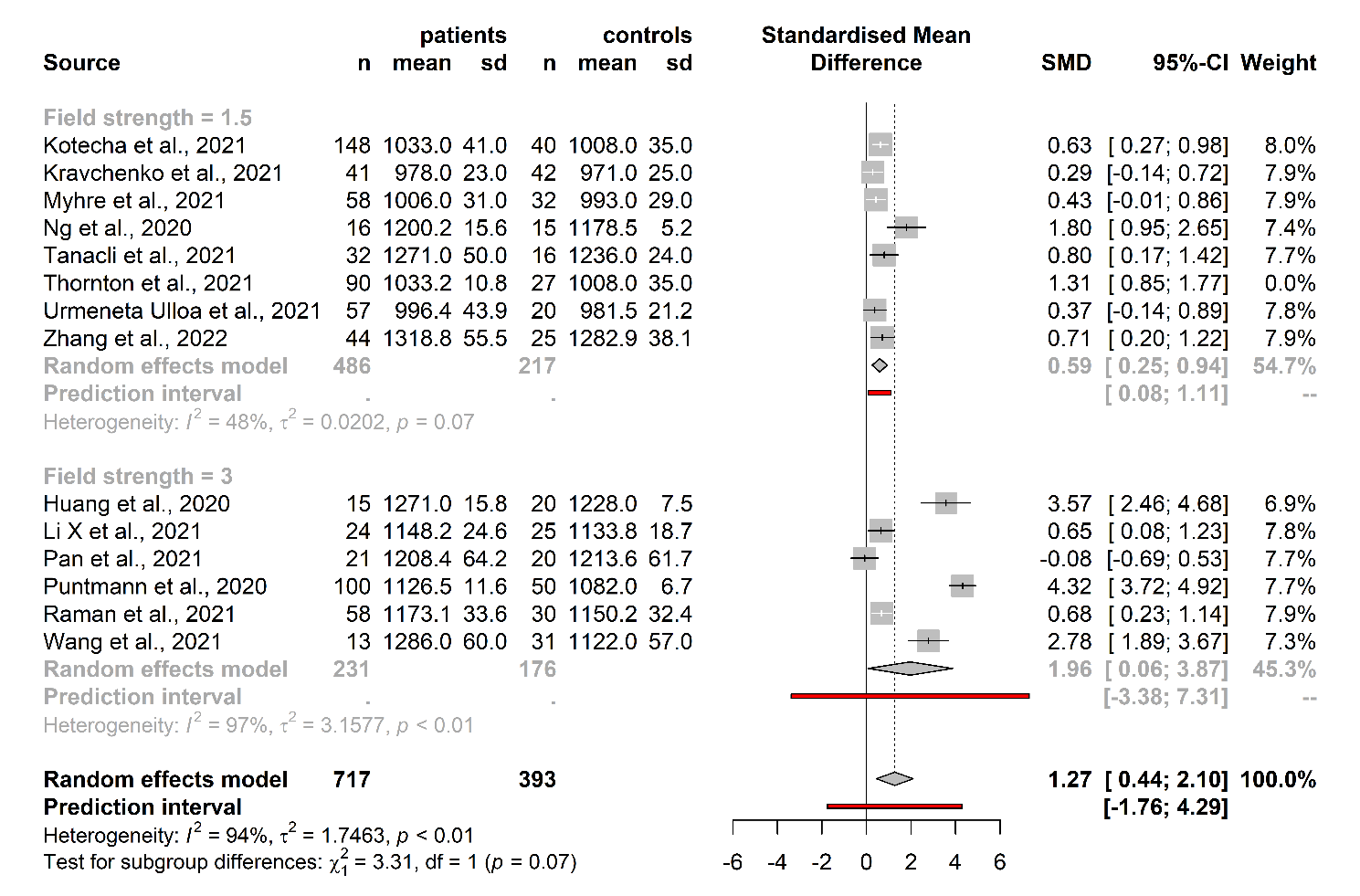


Supplementary Fig. 1. Effect size pooling of T1 native values in patients recovered from COVID-19. One report was identified as an outlier and excluded (Thornton *et al*.).

**
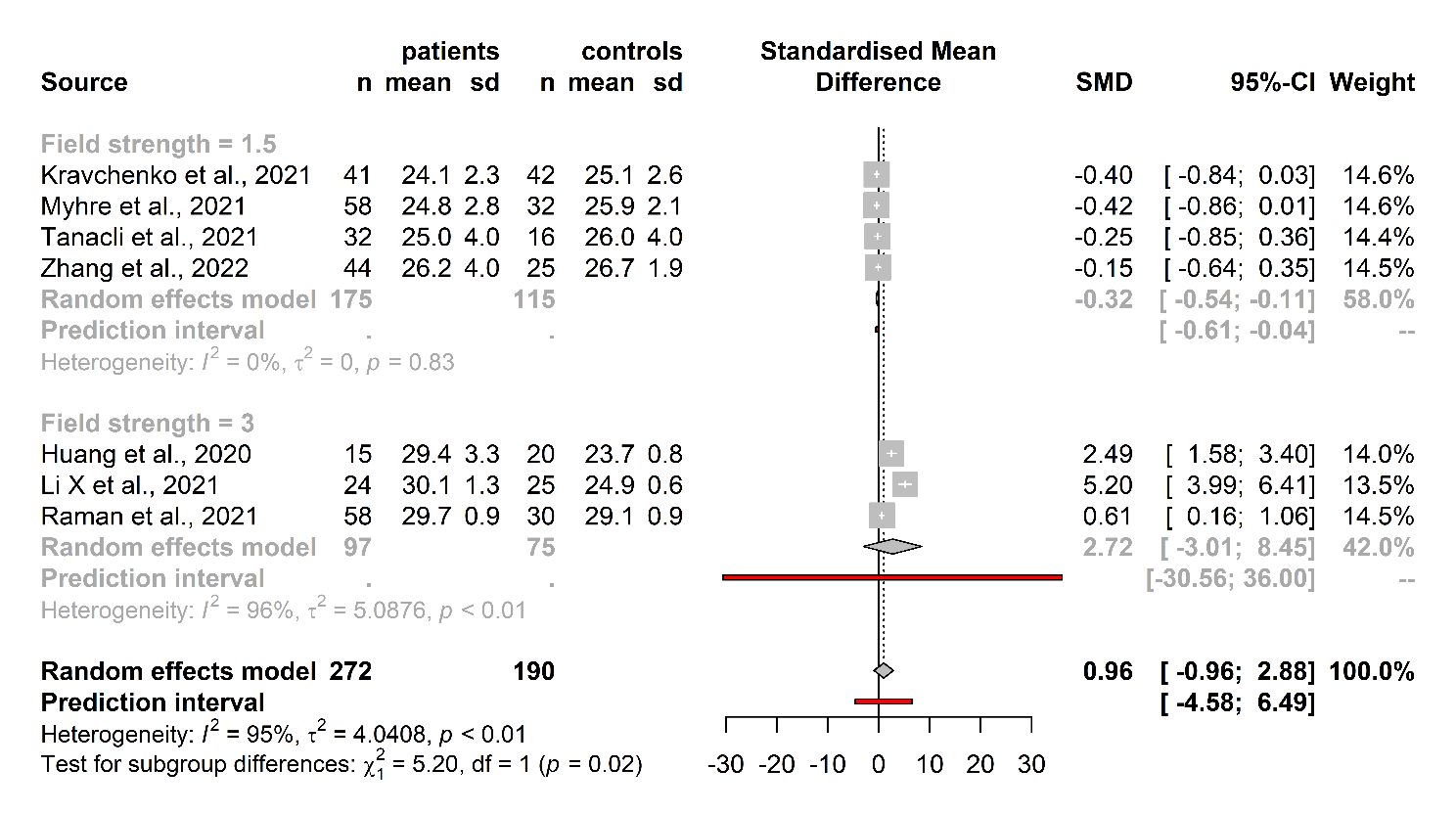
**

Supplementary Fig. 2. Effect size pooling of ECV values in patients recovered from COVID-19.


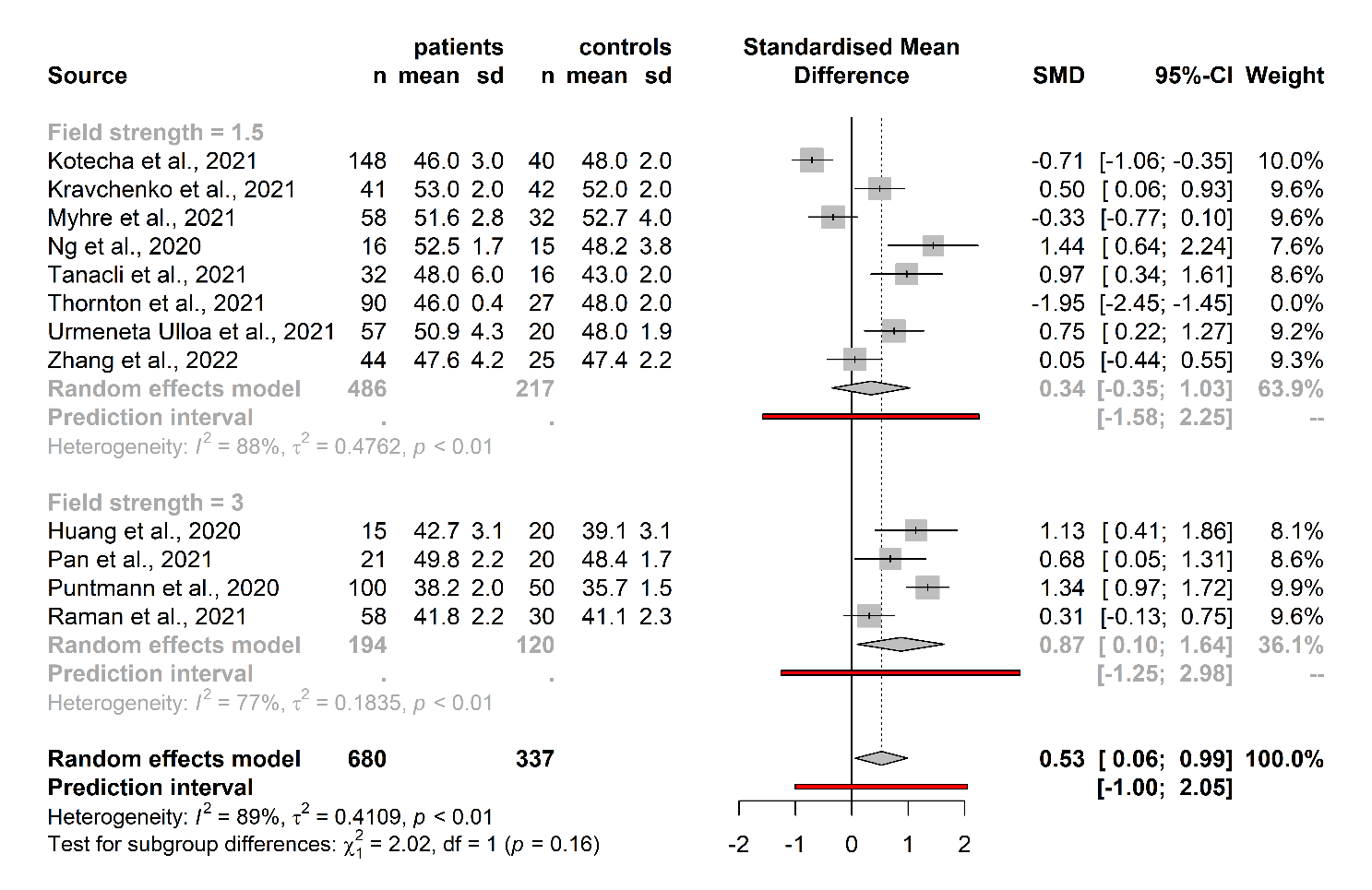


Supplementary Fig. 3. Effect size pooling of T2 values in patients recovered from COVID-19. One report was identified as an outlier and excluded (Thornton *et al*.).
